# Case Report: Ultra-early nusinersen initiation with pre-procedural spinal ultrasound-assisted intrathecal access in a symptomatic neonate with spinal muscular atrophy

**DOI:** 10.3389/fped.2026.1857511

**Published:** 2026-08-11

**Authors:** Ning Xie, Yan Sui, Yanyan Zhang, Leihong Zhang, Jiashan Li, Ying Sun, Xiuxiang Liu

**Affiliations:** 1 Neonatal Intensive Care Unit, Qingdao Women and Children’s Hospital, Qingdao, China; 2 Department of Medical Information, Qingdao Women and Children’s Hospital, Qingdao, China; 3 Department of Rehabilitation, Qingdao Women and Children’s Hospital, Qingdao, China; 4 Department of Genetic Testing Center, Qingdao Women and Children’s Hospital, Qingdao, China

**Keywords:** early treatment, lumbar puncture, nusinersen, spinal muscular atrophy, spinal ultrasound, symptomatic neonatal SMA

## Abstract

A full-term neonate presented at birth with generalized hypotonia, markedly reduced spontaneous movement, and tongue fasciculations. These findings raised early suspicion of an underlying severe neuromuscular disorder. Genetic testing confirmed homozygous deletion of SMN1 with two copies of SMN2 on day 5 of life, and intrathecal nusinersen was started on the same day. Because repeated lumbar puncture was required, spinal ultrasound was used before the first three intrathecal administrations during the neonatal period to evaluate lumbar anatomy and plan the puncture level and trajectory. This approach facilitated successful first-attempt intrathecal access during the early neonatal procedures. By day 68 of life, the infant had completed four loading doses without procedure-related complications. Motor function, assessed using the Children's Hospital of Philadelphia Infant Test of Neuromuscular Disorders (CHOP-INTEND), increased from 6 before treatment to 21 before the third dose and 30 before the fourth dose. Given the short follow-up period, these early changes should be interpreted cautiously. They are more likely to reflect early disease stabilization and preservation of residual motor function than reversal of established motor neuron loss. This case provides an individual-level real-world description of symptomatic neonatal spinal muscular atrophy treated within the first days of life after postnatal diagnosis. It also supports the feasibility of a structured ultrasound-assisted approach for early repeated intrathecal administration during the neonatal period.

## 1 Introduction

Spinal muscular atrophy (SMA) is a rare autosomal recessive neuromuscular disorder caused by mutations or deletions in the survival motor neuron 1 (SMN1) gene, leading to reduced levels of functional SMN protein and progressive degeneration of lower motor neurons. The clinical severity of SMA is primarily determined by the number of copies of the SMN2 gene (1). Infants with two SMN2 copies usually develop the most severe phenotype, characterized by early-onset hypotonia, muscle weakness, and progressive respiratory insufficiency if left untreated (2, 3). In the absence of disease-modifying treatment, most affected infants do not survive beyond 2 years of age. In recent years, disease-modifying therapies such as nusinersen have markedly improved survival and motor outcomes (4, 5). However, treatment response is strongly time dependent, and the greatest benefit has been observed in infants treated before symptoms appear (6).

With the increasing implementation of newborn screening (NBS), many patients can now be identified at a presymptomatic stage and treated early. However, symptomatic presentation in the neonatal period still occurs in routine clinical practice, especially in regions where screening is not universally available or where diagnosis may be delayed. These cases remain particularly challenging because the opportunity for intervention is narrow and clinical decisions must often be made rapidly after birth. Repeated intrathecal administration of nusinersen during this period adds a further procedural challenge, particularly in very small infants who require multiple early doses. Detailed descriptions of repeated neonatal intrathecal access, including the use of pre-procedural ultrasound planning, remain limited. Here, we report a symptomatic neonate with SMA who began nusinersen on day 5 of life after postnatal diagnosis. This case highlights a practical real-world scenario in which treatment could still be initiated during the earliest symptomatic neonatal stage and describes the use of pre-procedural spinal ultrasound to support repeated intrathecal access.

## 2 Case presentation

The study was approved by the Ethics Committee of the Women's and Children's Hospital affiliated to Qingdao University (No. 2026-80). Written informed consent was obtained from the patient's parents. This case report was prepared in accordance with the CARE guidelines.

### 2.1 Clinical findings

The patient was a male neonate born at 38 + 6 weeks of gestation via cesarean section due to fetal distress. He was the first live birth of a gravida 4, para 0 mother with a history of three unexplained early miscarriages. The pregnancy occurred naturally, and no prenatal genetic screening had been performed. Birth weight was 2,940 g (approximately the 20th percentile), head circumference was 34 cm (approximately the 40th percentile), and birth length was 50 cm (approximately the 50th percentile). Apgar scores were 6, 8, and 8 at 1, 5, and 10 minutes, respectively.

On day 1 of life, neurological examination revealed generalized hypotonia, markedly reduced spontaneous limb movements, and a frog-leg posture. The infant showed profound generalized weakness with minimal spontaneous antigravity movement in all extremities. His cry was weak, with reduced intensity and low pitch. Tremulous tongue movements consistent with fasciculations were observed, and deep tendon reflexes were absent. Despite these neuromuscular abnormalities, the infant remained alert and had stable cardiopulmonary status without clinical evidence of respiratory distress.

Initial laboratory evaluation showed a mildly elevated neuron-specific enolase level (34 ng/mL). Serum creatine kinase was elevated to 798.34 U/L on the first day of life but declined to 82.82 U/L after one week. Metabolic screening and the infection workup were unremarkable. By day 2 of life, hypotonia persisted without improvement and appeared disproportionate to the degree of perinatal compromise. The combination of preserved alertness, stable respiratory status, and persistent severe hypotonia raised strong suspicion of an underlying neuromuscular disorder. Electromyography was not performed during the neonatal period because an experienced neurophysiology team considered that the patient's very young age, small body size, and immature muscle development would limit the feasibility and interpretability of the examination. No additional neurophysiological testing, including nerve conduction studies, was performed, because nerve conduction studies were not available in our neonatal setting during the urgent diagnostic window. Anthropometric percentiles were interpreted according to the WHO Child Growth Standards for boys (7), using weight-for-age, length-for-age, and head circumference-for-age references.

### 2.2 Genetic diagnosis

**Figure 1:**
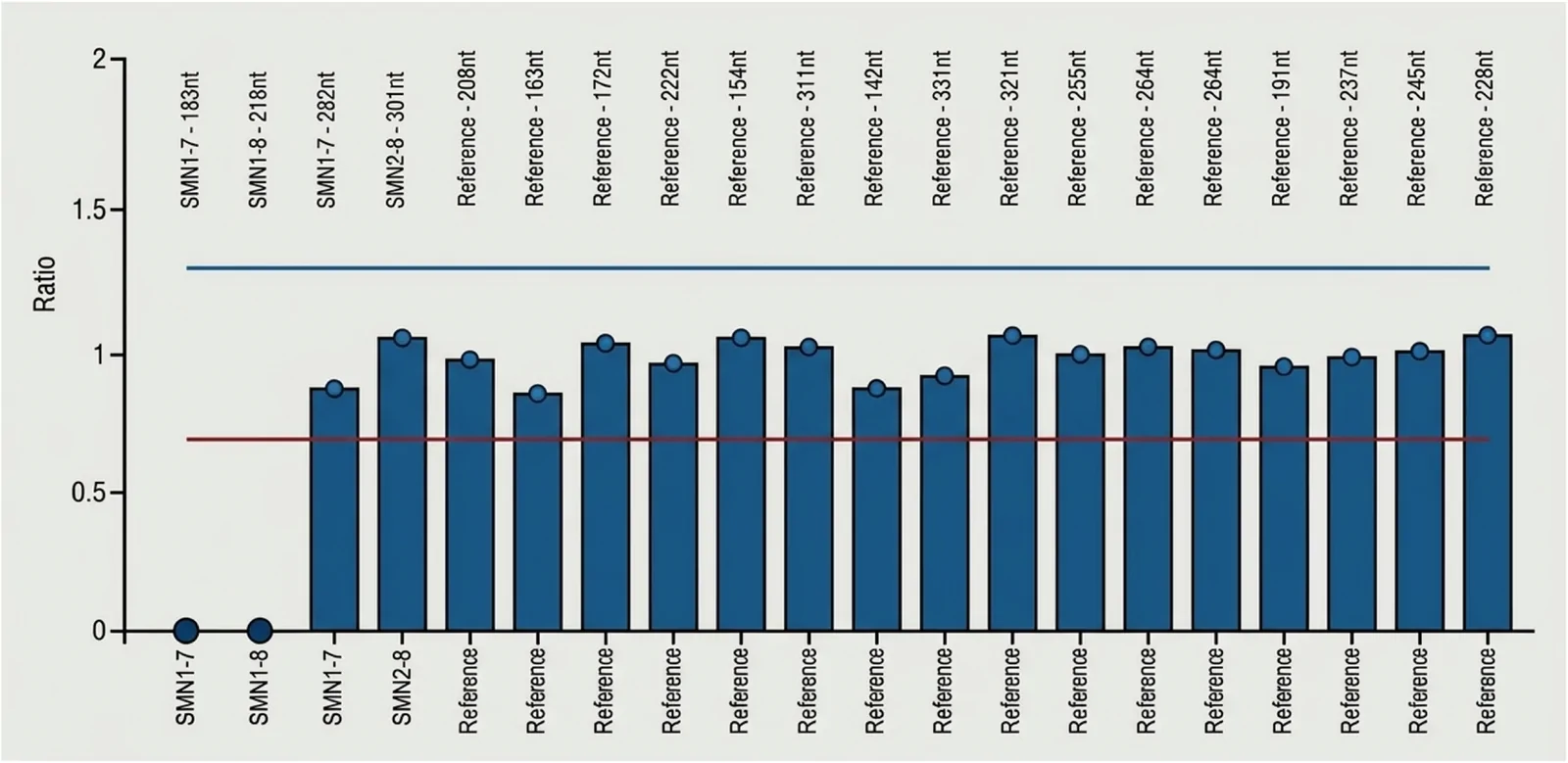
MLPA analysis of **SMN1** and **SMN2** copy number. Multiplex ligation-dependent probe amplification showed homozygous deletion of **SMN1** and two copies of **SMN2** exons 7 and 8, establishing the molecular diagnosis of spinal muscular atrophy.

Given the clinical presentation of severe hypotonia with preserved consciousness and stable respiration, a genetic etiology was strongly suspected. On day 3 of life, blood samples were obtained for multiplex ligation-dependent probe amplification (MLPA) and whole-exome sequencing. On day 4, the clinical team discussed the high suspicion of SMA and the potential need for immediate treatment with the parents, so that therapy could be initiated without delay once genetic confirmation became available. MLPA analysis subsequently confirmed homozygous deletion of SMN1 and the presence of two copies of SMN2 (exons 7 and 8) (Figure 1), establishing the diagnosis of SMA on day 5 of life, after which nusinersen was initiated. Subsequent whole-exome sequencing did not identify additional pathogenic variants associated with neonatal hypotonia.

### 2.3 Motor assessment

Motor function was assessed on day 5 of life, shortly before administration of the first dose of nusinersen, using the Children's Hospital of Philadelphia Infant Test of Neuromuscular Disorders (CHOP-INTEND) by a trained rehabilitation specialist. The baseline score was 6/64, indicating severe motor impairment, with minimal spontaneous limb movement and no antigravity control.

Follow-up assessment was performed on day 33 of life, before the third loading dose, by the same evaluator under comparable clinical conditions. The CHOP-INTEND score had increased to 21/64. Relative to baseline, the infant showed more spontaneous upper-limb movements, partial antigravity activity, and limited head rotation in the supine position.

A further assessment was completed on day 68 of life, before the fourth loading dose, and the CHOP-INTEND score was 30/64. Compared with the previous assessment, spontaneous upper-limb movement had further increased. Antigravity movement at the shoulder was observed, with the elbow lifting off the supporting surface, whereas previously only the hand and forearm could be raised. On the right side, weak grasping was observed together with lifting of the elbow from the supporting surface; on the left, finger flexion was present but no effective grasp was achieved. During rolling elicited from the legs and arms, the arm, shoulder, and trunk could be lifted from the supporting surface, although the movement remained posterior to the midline. In supported sitting with posterior trunk inclination, active elbow flexion was observed, and knee extension to approximately 30°–45° could be elicited with stimulation. In addition, several abilities showed qualitative improvement without a corresponding change in item score, including greater hip flexion-extension range, increased head rotation, longer maintenance of hip adduction, and a wider range of elbow movement.

Given the short observation interval and limited follow-up duration, these early changes should be interpreted cautiously. They are more appropriately regarded as a preliminary signal of disease stabilization and preservation of residual motor unit function than as restoration of previously lost motor neurons.

### 2.4 Pre-procedural spinal ultrasound-assisted lumbar puncture

Because nusinersen treatment requires repeated lumbar punctures, special attention is needed to avoid cumulative tissue injury from repeated punctures at or near the same site. In neonates, accurate pre-procedural anatomical assessment is therefore particularly important for selecting the safest puncture level and trajectory while minimizing injury to surrounding tissues.

**Figure 2:**
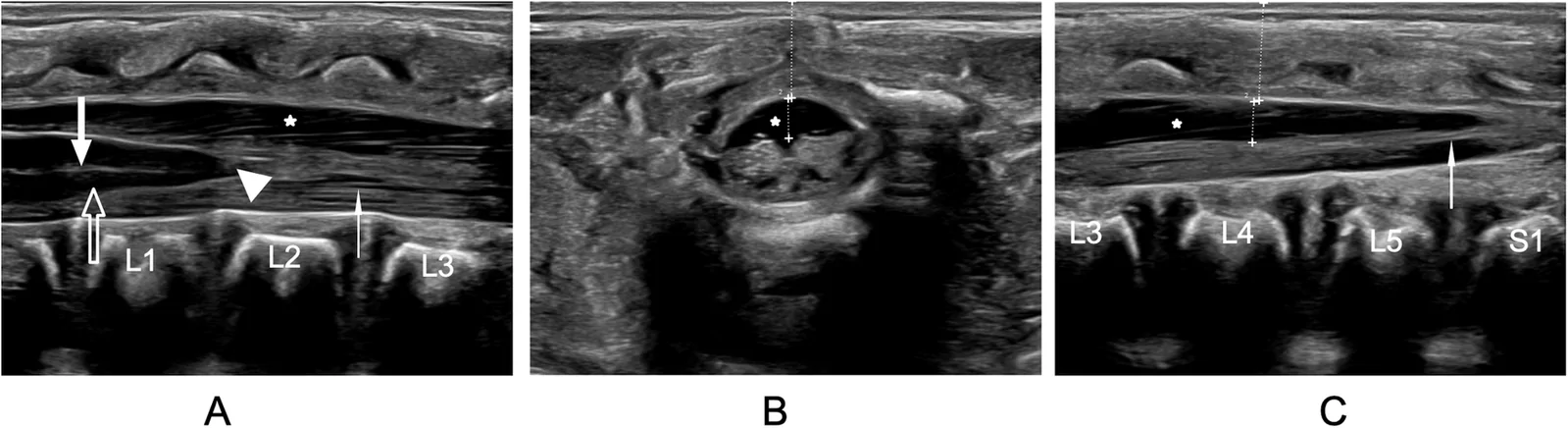
Pre-procedural spinal ultrasound for planning repeated intrathecal access. **(A)** Mid-sagittal sonographic view of the lumbar spine showing the vertebral levels (L1–L3). Within the spinal canal, the hypoechoic spinal cord (open arrow) contains the central echo complex (arrow), while the conus medullaris is identified distally (arrowhead). Caudal to the conus, the filum terminale (thin arrow) is visualized within the dural sac and is surrounded by the echogenic cauda equina nerve roots. This view allows identification of the caudal end of the spinal cord and helps avoid selection of an excessively high puncture level. **(B)** Axial sonographic view at the interlaminar level demonstrating the subarachnoid space (asterisks) and the intrathecal nerve roots. This view helps confirm midline spinal canal anatomy and the feasibility of safe intrathecal access. **(C)** Lower lumbar sagittal view showing the selected puncture level (L3–S1), the subarachnoid space (asterisk), and the filum terminale (thin arrow). The dotted line indicates measurement of the skin-to-dura distance, which was used to estimate the expected needle insertion depth and optimize the puncture trajectory.

Before each of the first three lumbar punctures during the neonatal period, the infant was placed in the lateral decubitus position on a firm, flat surface, with gentle flexion of the back and knees to widen the intervertebral spaces. In this position, the cauda equina nerve roots may layer dependently within the thecal sac, which can facilitate identification of the posterior cerebrospinal fluid space during pre-procedural ultrasound assessment. Pre-procedural spinal ultrasound was performed using a high-frequency linear transducer (Wisonic L15-4NB-H) to evaluate lumbar anatomy and plan an individualized intrathecal approach (Figure 2).

A mid-sagittal view was first obtained to identify the conus medullaris and assess the overall configuration of the lumbar spinal canal. The probe was then moved laterally to obtain parasagittal views for visualization of the posterior elements and adjacent paraspinal structures. Subsequently, axial scans were performed in a cranial-to-caudal sequence to identify the interlaminar spaces and dural sac at each vertebral level.

Based on these findings, the puncture site was selected according to two predefined criteria: (1) the widest interlaminar space, to maximize the acoustic window and improve procedural accessibility, and (2) the widest posterior subarachnoid space, to create a larger safety margin and reduce the risk of inadvertent injury to the cauda equina nerve roots or filum terminale. The planned needle angle and trajectory were determined by correlating the sagittal and axial planes to identify the safest and most direct path to the intrathecal space.

The findings from the first lumbar puncture are described here as a representative example of the ultrasound-based planning process. At the initial procedure, the L4–L5 interspace was selected for puncture, and the conus medullaris was identified at the L1–L2 level. The distance from the skin surface to the posterior margin of the subarachnoid space was approximately 9.4 mm. The centrally located intrathecal nerve roots and filum terminale were visualized at an additional depth of approximately 3.2 mm, indicating a narrow safety corridor for needle advancement after entry into the subarachnoid space. These measurements were used to guide controlled needle advancement and minimize the risk of inadvertent injury to neural structures. The same planning principles were applied before the subsequent two neonatal procedures, although minor variations in the measured parameters were observed between procedures.

Before each procedure, procedural sedation was provided with 10% chloral hydrate according to local practice. Topical 2.5% lidocaine cream was then applied to the intended puncture site approximately 60 minutes before the procedure and removed immediately before skin preparation. No general anesthesia or airway instrumentation was required. Under strict aseptic conditions, lumbar puncture was performed using a 22-gauge arterial puncture needle, which was selected because its length was appropriate for neonatal lumbar access, its rigidity facilitated controlled needle advancement, and its design allowed better control of cerebrospinal fluid outflow once the intrathecal space had been entered. Continuous cardiorespiratory monitoring was maintained throughout the procedure.

In this patient, pre-procedural spinal ultrasound facilitated individualized anatomical localization and consistent lumbar puncture planning during the first three intrathecal administrations in the neonatal period. Ultrasound was used for pre-procedural planning rather than real-time needle guidance. This approach may be particularly useful in neonates requiring repeated intrathecal therapy, in whom conventional landmark-based techniques are often limited by small anatomical structures and poorly defined surface landmarks.

### 2.5 Treatment and outcome

The first loading dose of nusinersen was administered on day 5 of life. The infant was discharged on day 10 with low-flow nasal cannula oxygen. Three days after discharge, supplemental oxygen was discontinued at home under pulse oximetry monitoring and remote medical guidance. The second and third loading doses were administered on days 19 and 33 of life, respectively. Each subsequent hospitalization lasted 2 days.

The first three loading doses were delivered in the neonatal unit using a pre-procedural spinal ultrasound-assisted approach. All three punctures were successful on the first attempt, and no procedure-related adverse events occurred. Brain MRI during the first hospitalization and routine cranial ultrasound before subsequent loading-dose administrations showed no evidence of hydrocephalus. During hospitalization, the infant remained hemodynamically stable and did not require invasive or non-invasive ventilatory support. Oral feeding was maintained, and breastfeeding continued. By the fourth hospitalization, body weight had increased to 4,500 g, head circumference to 37 cm, and length to 58 cm. Relative to birth, body weight had declined from approximately the 20th percentile to near the 5th percentile for age, and head circumference had decreased from approximately the 40th percentile to near the 5th percentile, whereas length remained around the 50th percentile.

CSF analysis was performed during the four intrathecal procedures. The CSF white blood cell counts were 1, 5, 6, and 3  ×  10^6^/L, respectively. CSF protein levels were 86.19, 55.65, 64.80, and 67.70 mg/dL, and CSF glucose levels were 2.97, 2.61, 2.77, and 2.72 mmol/L, respectively. No CSF findings suggested central nervous system infection during the reported period.

**Figure 3:**
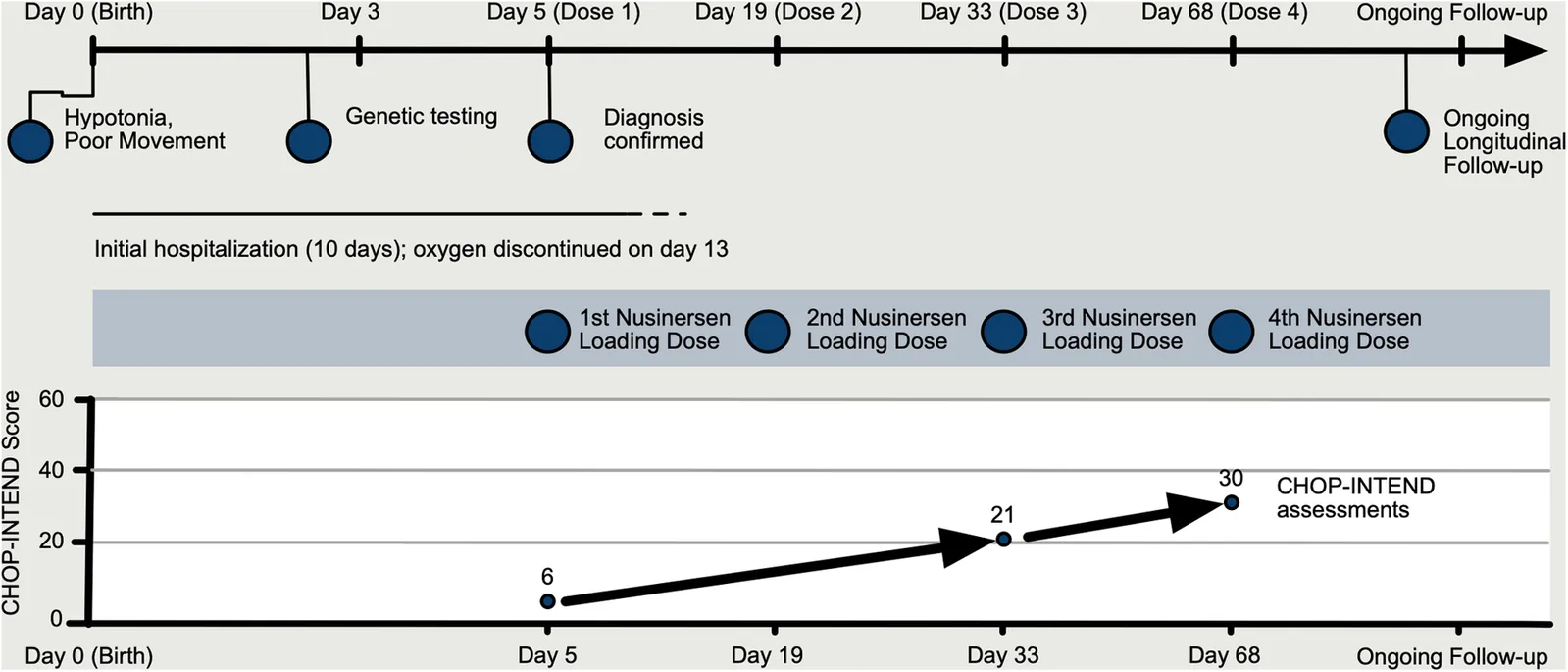
Clinical timeline of diagnosis and treatment. Timeline illustrating key clinical events from birth to day 68 of life, including genetic diagnosis, nusinersen administration, respiratory support changes, and serial CHOP-INTEND assessments.

After completion of the four loading doses, the infant entered the maintenance phase of nusinersen treatment. Structured follow-up assessments are planned before each maintenance dose, which is scheduled every 4 months. These assessments will include CHOP-INTEND, WHO motor milestones and the timing of newly achieved milestones, growth, respiratory status including respiratory support requirements and respiratory tract infections, feeding and swallowing status, and safety surveillance. Follow-up is ongoing, and the next structured assessment will be performed before the first maintenance dose. A detailed clinical timeline summarizing key diagnostic and therapeutic events is provided in Figure 3.

## 3 Discussion

SMA is a severe autosomal recessive neuromuscular disorder caused by biallelic pathogenic variants in SMN1, with an estimated incidence of approximately 1 in 6,000–10,000 live births and a carrier frequency of about 1:40–1:60 in the general population (8). Reduced SMN protein expression leads to progressive degeneration of lower motor neurons. In severe infantile-onset SMA, motor neuron loss and denervation begin very early, leaving a narrow therapeutic window for disease-modifying interventions. Previous clinical trials and newborn screening (NBS) cohorts have consistently demonstrated that presymptomatic treatment yields the most favorable outcomes, with many infants achieving motor milestones rarely observed in untreated patients (9–11). However, in real-world clinical practice, symptomatic presentation at birth remains a challenging scenario, particularly in regions where newborn screening or prenatal diagnosis is not routinely available.

In our setting, treatment selection was also influenced by drug availability and affordability. Nusinersen is an intrathecally administered antisense oligonucleotide that modifies SMN2 pre-mRNA splicing, whereas onasemnogene abeparvovec (Zolgensma) is an AAV9-based SMN1 gene replacement therapy (8). At the time of treatment, nusinersen was available through the national medical insurance system and could be initiated immediately after molecular confirmation, whereas onasemnogene abeparvovec had not yet received formal marketing approval in our country and was not available for routine clinical use. Therefore, nusinersen was selected as the immediately accessible disease-modifying therapy.

Although early nusinersen treatment has been reported in clinical trials and newborn screening cohorts, particularly in presymptomatic infants, published individual-level descriptions of symptomatic neonates treated within the first days of life after postnatal diagnosis remain limited. As summarized in Table 1, the available individual-level reports mainly include infants treated after prenatal diagnosis before overt symptoms appeared, together with a small number of symptomatic infants diagnosed after birth. Unver et al. (12) reported treatment initiation at 7 hours and 3 days of life in two prenatally diagnosed infants, both of whom were described as having favorable developmental outcomes during follow-up. In contrast, Nishino et al. (13) reported a symptomatic infant who began treatment at 55 days of life after postnatal diagnosis and later developed respiratory and feeding deterioration. In this selected comparison, our patient represents an uncommon real-world scenario: symptomatic neonatal SMA diagnosed after birth, with nusinersen started on day 5 of life. This position lies between presymptomatic very early treatment and more delayed postnatal intervention.

**Table 1:** Selected published reports of very early nusinersen initiation in SMA.

Study	SMN2 copies	Symptomatic at first dose	Age at first dose	Diagnostic setting/baseline features	Follow-up/reported outcome
Unver et al.	2	No	7 h	Prenatal diagnosis; absent DTRs	Normal development reported at 13 months
Unver et al.	3	No	3 days	Prenatal diagnosis; no overt SMA manifestations	Normal development reported at almost 6 years
Nishino et al.	2	Yes	55 days	Postnatal diagnosis; hypotonia, absent DTRs	Respiratory and feeding deterioration reported at 6 months
Present case	2	Yes	5 days	Postnatal diagnosis; generalized hypotonia, markedly reduced spontaneous movement, weak cry, tongue fasciculations, absent DTRs	Four loading doses completed by day 68; CHOP-INTEND increased from 6 to 30; no procedure-related adverse events

The table includes selected published individual-level neonatal cases of very early nusinersen initiation with sufficient clinical detail for direct comparison. DTRs, deep tendon reflexes; SMA, spinal muscular atrophy.

In our patient, the CHOP-INTEND score increased from 6/64 before treatment to 21/64 before the third loading dose and 30/64 before the fourth dose on day 68 of life. Serial assessments also documented modest changes in motor performance, including greater spontaneous upper-limb movement and the emergence of proximal antigravity activity. These early findings should be interpreted cautiously. Given the short follow-up period, they are more appropriately regarded as an early indication of disease stabilization and preservation of residual motor function rather than reversal of established motor neuron loss. This point is particularly important in neonatal SMA, in which irreversible motor neuron loss is believed to begin very early. At the same time, longitudinal natural history data in untreated SMA type 1 show no sustained improvement in CHOP-INTEND scores over time, with neonatal-onset infants showing low baseline scores and rapid decline (14). This suggests that even short-term stabilization may be clinically relevant in this setting.

In addition to treatment timing, this case highlights a practical issue in neonatal care: repeated intrathecal administration in a very small infant. Lumbar puncture in this population is technically challenging because anatomical structures are small and surface landmarks are often poorly defined. Ultrasound-assisted lumbar puncture is already used in neonatal and infant practice. A systematic review and meta-analysis of randomized or quasi-randomized trials in neonates and infants reported that ultrasound imaging significantly reduced the risk of traumatic lumbar puncture, although the reduction in lumbar puncture failure was not statistically significant (15). In our patient, pre-procedural spinal ultrasound was used as a planning tool rather than as real-time needle guidance. It helped identify the conus medullaris, select the puncture level, assess the posterior cerebrospinal fluid space, and provide a reference for controlled needle advancement as the intrathecal space was approached. In this context, the value of ultrasound was mainly practical: it supported repeated intrathecal nusinersen administration in a very small symptomatic neonate by allowing a consistent puncture plan across the loading phase. Expert recommendations also support the use of spinal ultrasound in neonates when landmarks are difficult to define (16).

However, procedural safety extends beyond puncture success. A recent report highlighted that infants with SMA may require careful neurological surveillance and individualized anesthetic planning during the course of intrathecal nusinersen treatment, including the possibility of hydrocephalus requiring ventriculoperitoneal shunting (17). Accordingly, head circumference, anterior fontanelle status, neurological signs, and cranial ultrasound findings will be monitored during follow-up, with further neuroimaging considered if clinical or ultrasonographic concerns arise.

Although early motor changes were observed, somatic growth was not uniform during short-term follow-up. By day 68 of life, body weight and head circumference had both fallen to near the 5th percentile, whereas linear growth appeared relatively preserved, with length remaining around the 50th percentile. Several factors may have contributed to this pattern. Although oral feeding was maintained, sucking strength appeared limited on clinical observation, and feeding efficiency may therefore have been suboptimal. In addition, reduced spontaneous movement, low muscle mass related to the underlying disease, and increased energy expenditure associated with neuromuscular weakness may also have affected early weight gain. These observations suggest that short-term motor stabilization does not necessarily translate into parallel improvement in growth and highlight the importance of ongoing nutritional and developmental monitoring in symptomatic neonatal SMA.

Several limitations should be acknowledged. As a single case, the findings cannot be generalized, particularly given the heterogeneity of SMA phenotypes and the influence of SMN2 copy number on disease severity. In addition, the duration of follow-up remains limited, and long-term outcomes, including motor milestone acquisition and respiratory function, have yet to be determined. Furthermore, although early improvement in CHOP-INTEND score was observed, this finding should be interpreted cautiously in the absence of a comparator group. Finally, the successful use of pre-procedural spinal ultrasound-assisted lumbar puncture planning reflects a single-center experience and may depend on operator expertise.

Despite these limitations, this case provides clinically relevant insight into the feasibility of early intervention in symptomatic neonatal SMA and highlights the importance of rapid diagnosis and timely treatment in real-world clinical settings. These findings may be particularly relevant for healthcare systems where access to newborn screening or prenatal diagnosis remains limited.

## 4 Conclusion

Overall, this case provides a real-world description of very early nusinersen initiation in symptomatic neonatal SMA after postnatal diagnosis. It suggests that a clinically meaningful treatment window may still remain in the earliest symptomatic stage, even when symptoms are evident at birth. In this infant, rapid postnatal diagnosis enabled nusinersen initiation on day 5 of life, and early intrathecal administrations during the neonatal period were facilitated by a structured pre-procedural spinal ultrasound approach. Longer follow-up is needed to determine the durability of motor and respiratory outcomes.

## Data Availability

The original contributions presented in the study are included in the article; further inquiries can be directed to the corresponding author.

## References

[1] Butchbach MER. Genomic variability in the survival motor neuron genes (SMN1 and SMN2): implications for spinal muscular atrophy phenotype and therapeutics development. Int J Mol Sci. (2021) 22(15):7896. 10.3390/ijms22157896 34360669 PMC8348669

[2] Nmer S Trhanint S Sayel H Chaouki S Bouguenouch L Ouldim K. First combined analysis of SMN1, SMN2, and NAIP copy numbers in Moroccan SMA patients and their correlation with disease severity. Mol Genet Metab Rep. (2026) 46:101299. 10.1016/j.ymgmr.2026.101299 41773285 PMC12950346

[3] Chakraborty S Singh A Perveen S Chowdhury MR Ali S Gupta N. Correlation between neuronal apoptosis inhibitory protein (NAIP), SMN2, and SMA phenotypes: a tertiary care centre experience from India. Am J Med Genet A. (2025) 197(7):e64057. 10.1002/ajmg.a.64057 40099840

[4] Finkel RS Mercuri E Darras BT Connolly AM Kuntz NL Kirschner J. Nusinersen versus sham control in infantile-onset spinal muscular atrophy. N Engl J Med. (2017) 377(18):1723–32. 10.1056/NEJMoa1702752 29091570

[5] Mercuri E Darras BT Chiriboga CA Day JW Campbell C Connolly AM. Nusinersen versus sham control in later-onset spinal muscular atrophy. N Engl J Med. (2018) 378(7):625–35. 10.1056/NEJMoa1710504 29443664

[6] Cooper K Nalbant G Sutton A Harnan S Thokala P Chilcott J. Systematic review of presymptomatic treatment for spinal muscular atrophy. Int J Neonatal Screen. (2024) 10(3):56. 10.3390/ijns10030056 39189228 PMC11348213

[7] World Health Organization. WHO Child Growth Standards: Length/height-for-age, Weight-for-age, Weight-for-length, Weight-for-height and Body Mass index-for-age: Methods and Development. Geneva: World Health Organization (2006).

[8] Mercuri E Sumner CJ Muntoni F Darras BT Finkel RS. Spinal muscular atrophy. Nat Rev Dis Primers. (2022) 8(1):52. 10.1038/s41572-022-00380-8 35927425

[9] Crawford TO Swoboda KJ De Vivo DC Bertini E Hwu W-L Finkel RS. Continued benefit of nusinersen initiated in the presymptomatic stage of spinal muscular atrophy: 5-year update of the NURTURE study. Muscle Nerve. (2023) 68(2):157–70. 10.1002/mus.27853 37409780

[10] Kariyawasam DS D’Silva AM Sampaio H Briggs N Herbert K Wiley V. Newborn screening for spinal muscular atrophy in Australia: a non-randomised cohort study. Lancet Child Adolesc Health. (2023) 7(3):159–70. 10.1016/S2352-4642(22)00342-X 36669516

[11] Schwartz O Vill K Pfaffenlehner M Behrens M Weiß C Johannsen J. Clinical effectiveness of newborn screening for spinal muscular atrophy: a nonrandomized controlled trial. JAMA Pediatr. (2024) 178(6):540–7. 10.1001/jamapediatrics.2024.0492 38587854 PMC11002769

[12] Ünver O Çelik T Memişoğlu A Büyükbayrak EE Tülin Şimşek F Öztürk G. The outcome of two SMA cases treated with nusinersen at seven hours and at three days of life: the earliest ever. Neuromuscul Disord. (2022) 32(7):575–7. 10.1016/j.nmd.2022.06.002 35752576

[13] Nishino E Namatame N Kawano O Egawa K Ogasawara T Kaneda M. A neonatal case of spinal muscular atrophy type I treated with nusinersen from 55 days of age. No to Hattatsu. (2020) 52(1):38–40. 10.11251/ojjscn.52.38

[14] Mercuri E Lucibello S Perulli M Coratti G De Sanctis R Pera MC. Longitudinal natural history of type I spinal muscular atrophy: a critical review. Orphanet J Rare Dis. (2020) 15(1):84. 10.1186/s13023-020-01356-1 32248834 PMC7132885

[15] Olowoyeye A Fadahunsi O Okudo J Opaneye O Okwundu C. Ultrasound imaging versus palpation method for diagnostic lumbar puncture in neonates and infants: a systematic review and meta-analysis. BMJ Paediatr Open. (2019) 3(1):e000412. 10.1136/bmjpo-2018-000412 30957031 PMC6422243

[16] American Institute of Ultrasound in Medicine, American College of Radiology, Society for Pediatric Radiology, Society of Radiologists in Ultrasound. AIUM practice guideline for the performance of an ultrasound examination of the neonatal spine. J Ultrasound Med. (2012) 31(1):155–64. 10.7863/jum.2012.31.1.155 22215784

[17] Nastasovic T Micovic M Milenovic M Minic M Bojic S. Anesthesia in infant with spinal muscular atrophy type 1 for ventriculoperitoneal shunt placement. Srp Arh Celok Lek. (2025) 153(7–8):396–9. 10.2298/sarh250112051n

